# Combining Partial True Discovery Guarantee Procedures

**DOI:** 10.1002/bimj.202300075

**Published:** 2024-07-02

**Authors:** Ningning Xu, Aldo Solari, Jelle J. Goeman

**Affiliations:** ^1^ Department of Biomedical Data Sciences Leiden University Medical Center Leiden The Netherlands; ^2^ Department of Economics Management and Statistics University of Milano‐Bicocca Milan Italy; ^3^ Department of Economics Ca' Foscari University of Venice Venice Italy

**Keywords:** false discovery proportion, multiple testing, simultaneous inference

## Abstract

Closed testing has recently been shown to be optimal for simultaneous true discovery proportion control. It is, however, challenging to construct true discovery guarantee procedures in such a way that it focuses power on some feature sets chosen by users based on their specific interest or expertise. We propose a procedure that allows users to target power on prespecified feature sets, that is, “focus sets.” Still, the method also allows inference for feature sets chosen post hoc, that is, “nonfocus sets,” for which we deduce a true discovery lower confidence bound by interpolation. Our procedure is built from partial true discovery guarantee procedures combined with Holm's procedure and is a conservative shortcut to the closed testing procedure. A simulation study confirms that the statistical power of our method is relatively high for focus sets, at the cost of power for nonfocus sets, as desired. In addition, we investigate its power property for sets with specific structures, for example, trees and directed acyclic graphs. We also compare our method with AdaFilter in the context of replicability analysis. The application of our method is illustrated with a gene ontology analysis in gene expression data.

## 1 Introduction

### 1.1 Background

In multiple hypotheses testing, a recent approach is simultaneous (and thus post hoc) inference. It allows researchers to examine the data and obtain valid true discovery proportion (TDP) guarantees, that is, a lower confidence bound for TDP, simultaneously for all possible subsets of hypotheses (Blanchard, Neuvial, and Roquain 2020; Genovese and Wasserman 2006; Goeman and Solari 2011; Goeman, Hemerik, and Solari 2021). True discovery guarantee procedures have been applied in genetics (Ebrahimpoor et al. 2020; Ebrahimpoor and Goeman 2021) and brain imaging (Andreella et al. 2023; Blain, Thirion, and Neuvial 2022; Rosenblatt et al. 2018).

True discovery guarantee procedures, however, can have very different power properties depending on the choice of testing methods, for example, some methods are powerful for sparse alternatives and some for dense alternatives (Goeman, Hemerik, and Solari 2021; Tian et al. 2023; Vesely, Finos, and Goeman 2023). Additionally, the power of the procedures depends on the structure of the sets to be tested, for example, tree structures (Bogomolov et al. 2021) or directed acyclic graphs (DAGs; Meijer and Goeman 2015). It is a challenging problem to construct a true discovery guarantee procedure that has good putative properties, targeting its power to specific sets of interest.

The goal of this paper is to design a true discovery guarantee procedure that targets its power towards a collection of a priori chosen feature sets. Existing procedures that direct their power towards chosen sets include methods to control family‐wise error rate (Goeman and Mansmann 2008; Meinshausen 2008) or to control the False Discovery Proportion (FDP) with nested or tree‐structured reference families (Blanchard, Neuvial, and Roquain 2020; Durand et al. 2020). Our novel approach is similar in spirit to the focus‐level procedure of Goeman and Mansmann (2008) but improves upon that method using the techniques of Blanchard, Neuvial, and Roquain (2020) and Goeman, Hemerik, and Solari (2021).

We use prespecified feature sets as a reference family, which we call “focus sets.” Within each focus set, we suppose that a partial true discovery guarantee procedure is given, which will be used as building blocks of our post hoc true discovery guarantee procedure. The procedure is general and allows for any partial true discovery guarantee procedure on the focus set. As intended, the statistical power of our method is relatively high for focus sets, though this increased power comes at the cost of power for nonfocus sets.

Closed testing has been proven to be the only admissible way to achieve a simultaneous true discovery guarantee procedure, that is, alternative procedures either are equivalent to closed testing or can be uniformly improved by it (Goeman, Hemerik, and Solari 2021). We, therefore, show that the proposed procedure is a shortcut to a closed testing procedure, and we construct the local test of this closed testing procedure explicitly. The worst‐case computation time of closed testing is exponential in general, but we present several shortcuts which can dramatically reduce computation time. These shortcuts retain the true discovery guarantee but come at the cost of some reduction in power. The shortcuts assume that efficient algorithms are available for the partial true discovery guarantee procedures on the focus sets (Brannath and Bretz 2010; Blanchard, Neuvial, and Roquain 2020; Dobriban 2020; Durand et al. 2020; Gou et al. 2014; Goeman et al. 2019; Tian et al. 2023; Vesely, Finos, and Goeman 2023).

To support our theoretical results, we demonstrate the targeted effectiveness of our procedure on focus sets using artificial data. We compare our method with the methods for which hypotheses are in a DAG (Meijer and Goeman 2015) and in a tree (Durand et al. 2020). We make no assumptions about the focus sets; they can be disjoint or overlapped, for which the power difference is presented by using a toy example in the Supporting Information. We also investigate in some depth an application to replicability analysis, in which the focus sets can be taken as disjoint and the method is simplified. For this case, we show a qualitative and quantitative improvement of our approach over AdaFilter (Wang et al. 2022). The general method is illustrated with a gene ontology analysis in gene expression data in the Supporting Information.

### 1.2 Notation and Preliminaries

We assume that data are distributed according to some unknown probability distribution P∈Ω. Let W be the whole feature set and ${\left(H_{s}\right)}_{s\in W}$ be the family of null hypotheses to be tested corresponding to |W| features, where |·| denotes the cardinality of a set. We have $H_{s}\subseteq \Omega$ for all s∈W, and a hypothesis H is true if and only if P∈H. The set of true null hypotheses in W is denoted by $W_{0}=\left\{s\in W:P\in H_{s}\right\}$, and $W_{1}=W\setminus W_{0}$ is the set of false hypotheses. For any feature set I⊆W, the true discoveries in I are $W_{1}\cap I$ and the false discoveries are $W_{0}\cap I$.

We define intersection hypotheses as $H_{I}=\cap_{s\in I}H_{s}$, for any $I\in 2^{W}$, where $2^{W}$ denotes the family of all possible subsets of W. An intersection hypothesis $H_{I}$ is true if and only if all individual hypotheses $H_{s},s\in I$ are true. For the special case I=∅, we always have $H_{\emptyset }=\Omega$.

Suppose that certain subsets of the hypotheses are a priori of particular interest, which we call focus sets. These are chosen based on practitioner's experience or background knowledge. Let the collection of focus sets be $F=\{F_{1},\text{…},F_{m}\}$, where each $F_{i}\subseteq W$, 1≤i≤m. We assume that the focus sets are independent of the data used for testing, so that they can be considered fixed. We do not impose any further restrictions on the focus sets, which can overlap or be disjoint with each other.

As defined in Goeman, Hemerik, and Solari (2021), a true discovery guarantee procedure $d^{\alpha }$ provides a lower confidence bound for the number of true discoveries in any set of interest, with confidence level 1−α, that is, for any S⊆W,

(1) $$P(d^{\alpha }\left(S\right)\leq |S\cap W_{1}|\;\text{for all}\;S\in 2^{W})\geq 1-\alpha .$$

In a similar way, a partial true discovery guarantee procedure is defined for a subfamily of the hypotheses. Without loss of generality, a partial procedure $d_{i}^{\alpha }$ for the subfamily $2^{F_{i}}$ satisfies that

(2) $$P(d_{i}^{\alpha }\left(S\right)\leq |S\cap W_{1}|\;\text{for all}\;S\in 2^{F_{i}})\geq 1-\alpha .$$

## 2 Combining Partial True Discovery Guarantee Procedures

### 2.1 Bonferroni‐Based Combination

To control the error rate, we perform each partial true discovery guarantee procedure at the Bonferroni corrected significance level, that is, α/m, for the focus sets. These partial procedures are then used as building blocks to calculate the true discovery lower bounds for any set chosen after seeing the data. We will initially focus exclusively on finding the true discovery bound in set S for the case that $S=F_{i}$, for some i, only, extending to other S later in this section.

We start by defining a trivial procedure $d^{\left(0\right)}$ that uses only information from focus sets and only gives nontrivial results for focus sets, that is,

(3) $$d^{\left(0\right)}\left(S\right)=\left\{\begin{array}{c} d_{i}^{\alpha /m},\exists F_{i}\in F,S=F_{i},\\ 0,\text{otherwise}, \end{array}\right.$$

where we use the shorthand $d_{i}^{\alpha /m}=d_{i}^{\alpha /m}\left(F_{i}\right)$. The true discovery guarantee of $d^{\left(0\right)}$ follows directly from Equation (2).

Following Goeman, Hemerik, and Solari (2021), the trivial procedure can be improved by interpolation. After interpolation, the procedure may give nontrivial bounds for nonfocus sets as well as focus sets. The interpolated version of the lower bound is defined as:

(4) $$d^{\left(k+1\right)}\left(S\right)=\max_{U\in 2^{W}}\{d^{\left(k\right)}\left(U\right)-|U\setminus S|+d^{\left(k\right)}\left(S\setminus U\right)\}.$$

The rationale of the interpolation is that if the number of true discoveries in U exceeds |U∖S| the remainder must be in S. Interpolation is especially useful if S has a large overlap with some U for which $d^{\left(k\right)}\left(U\right)$ is large. The interpolated procedure may improve upon the original procedure for focus sets as well. It always gives at least as good bound as the procedure it interpolates. We have

(5) $$d^{\left(k+1\right)}\left(S\right)\geq d^{\left(k\right)}\left(S\right).$$

Moreover, by Goeman, Hemerik, and Solari (2021), Lemma 2, $d^{\left(k+1\right)}$ is a true discovery guarantee procedure if $d^{\left(k\right)}$ is. The interpolated procedures can be improved again by another round of interpolation. The procedure that cannot be further improved by interpolation is called a coherent procedure (Goeman, Hemerik, and Solari 2021). Let r be a number of interpolations after which the procedure cannot be improved anymore, that is, $d^{\left(r\right)}\left(S\right)=d^{\left(r+1\right)}\left(S\right)$.

We illustrate the process of interpolation using a toy example with four features. Suppose that we have two focus sets: $F_{1}=\left\{1,2\right\}$ and $F_{2}=\left\{2,4\right\}$ with $d_{1}^{\alpha /m}\left(F_{1}\right)=1,d_{2}^{\alpha /m}\left(F_{2}\right)=2$. The lower bound of true discoveries in any nonfocus set, say {2,3}, can be computed based on Equation (4), that is,

$$\begin{array}{c} d^{\left(1\right)}\left(\left\{2,3\right\}\right)=\max\left\{\begin{array}{c} d^{\left(0\right)}\left(\left\{1,2\right\}\right)-\left|\left\{1\right\}\right|+d^{\left(0\right)}\left(\left\{3\right\}\right)=0,\;\text{for}\;F_{1},\\ d^{\left(0\right)}\left(\left\{2,4\right\}\right)-\left|\left\{4\right\}\right|+d^{\left(0\right)}\left(\left\{3\right\}\right)=1,\;\text{for}\;F_{2},\\ 0,\;\text{for all nonfocus sets.} \end{array}\right. \end{array}$$

Table 1 summarizes the improvement by interpolation for all feature sets. We see that $d^{\left(1\right)}$ is a coherent procedure since it cannot be improved further.

**TABLE 1:** Improvement to $d^{\left(0\right)}$ by interpolation. After two rounds, it converges to closed testing.

Feature set	$d^{\left(0\right)}$	$d^{\left(1\right)}$	$d^{\left(2\right)}$
∅	0	0	0
{1}	0	0	0
{2}	0	1	1
{3}	0	0	0
{4}	0	1	1
{12}	1	1	1
{13}	0	0	0
{14}	0	1	1
{23}	0	1	1
{24}	2	2	2
{34}	0	1	1
{123}	0	1	1
{124}	0	2	2
{134}	0	1	1
{234}	0	2	2
{1234}	0	2	2

*Note*: The bold feature sets represent the focus sets.

### 2.2 Holm‐Based Combination

To improve upon the Bonferroni‐based combinations of the previous section, we can use a variant of the procedure of Holm (1979), following the same principle that was used in the focus‐level procedure of Goeman and Mansmann (2008).

Call h “Holm's factor” and initialize it to h=m. For every focus set $F_{i}$ for which the partial true discovery procedure rejects all hypotheses, that is, $d_{i}^{\alpha /h}\left(F_{j}\right)=\left|F_{i}\right|$, Holm's factor is reduced by one. Next, $d_{j}^{\alpha /h}\left(F_{j}\right)$ is recalculated for the remaining focus sets using the updated h. These steps are repeated until no new completely rejected focus sets are found, which mostly happens in a handful of steps. This results in Algorithm 1.

ALGORITHM 1 **Partial true discovery procedures with Holm's procedure.**

**Table:** 

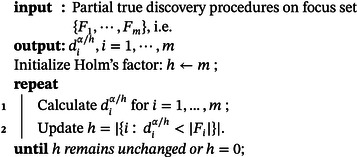

In the following sections, we prefer the Holm‐based combination. However, we may sometimes revert to the Bonferroni‐based combination to improve computational speed at the cost of some power.

## 3 Fast Greedy Algorithm for Interpolation

We note that the new procedure optimizes over exponentially many sets in (4). In practice, however, it is hardly necessary to try out all $U\in 2^{W}$. We note that discoveries in S come through focus sets exclusively and predominantly through focus sets with many discoveries and a large overlap with S. Therefore, we can approximate $d^{\left(r\right)}\left(S\right)$ with a greedy algorithm that chooses focus sets based on the number of discoveries they infer about S. This procedure is given in Algorithm 2. It retains control but may sacrifice some power.

ALGORITHM 2 **Greedy algorithm for interpolations.**

**Table:** 

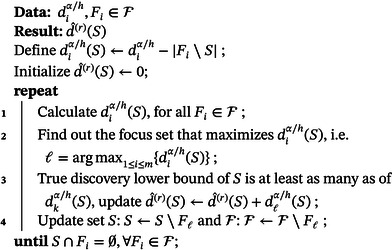

It is obvious that the number of iterations in the above algorithm is no more than m. The number is small if the set is less likely to overlap with the focus sets and close to m if the set is more likely a union of the focus sets.

The following Lemma 3.1 shows that ${\hat{d}}^{\left(r\right)}\left(S\right)$ is a shortcut to $d^{\left(r\right)}\left(S\right)$, the proof of which can be found in the Supporting Information.

Lemma 3.1 ${\hat{d}}^{\left(r\right)}\left(S\right)\leq d^{\left(r\right)}\left(S\right)$ for all S⊆W.

Finally, if the partial true discovery guarantee procedures are a computational bottleneck, we may want to avoid updating Holm's factor too often in Algorithm 1. For this situation, we note that error control is retained if Algorithm 1 is stopped early. In the most extreme case, we may not update Holm's factor h at all but keep h=m.

## 4 A Full Closed Testing Procedure

As argued by Goeman, Hemerik, and Solari (2021), the closed testing procedure is the only admissible procedure for true discovery guarantee: all other procedures are either equivalent to closed testing or can be improved by it. In this section, we construct an admissible closed testing procedure that uniformly improves upon the true discovery guarantee procedure constructed above. The purpose of constructing this procedure is to investigate the “computational gaps,” that is, the places where the procedure we have described above sacrifices power for computational reasons, compared to the full closed testing procedure.

### 4.1 The Closed Testing Procedure

To construct the closed testing procedure, we propose a local test for every hypothesis $H_{S},S\subseteq W$, defined as

(6) $$\phi \left(S\right)=\left\{\begin{array}{cc} 1, & \text{if}\;d_{i}^{\alpha /m_{S}}\left(F_{i}\cap S\right)>0\;\text{for at least one}\;1\leq i\leq m,\\ 0, & \text{otherwise}, \end{array}\right.$$

where $m_{S}=\#\left\{i:F_{i}\cap S\neq \emptyset \right\}$ is the number of focus sets that have a nonempty intersection with S. ϕ(S)=1 indicates the rejection of $H_{S}$, when at least one partial true discovery guarantee procedure, at level $\alpha /m_{S}$, reports a positive number of true discoveries in S. The following lemma asserts that the local test ϕ(S) controls type I error at α.

Lemma 4.1 If $P\in H_{S}$, then P(ϕ(S)=1)≤α.

Based on the local test ϕ, we can construct the closed testing procedure, which fulfills (1). It is given by

(7) $$d\left(I\right)=\min_{J\subseteq I}\left\{|I\setminus J|:\psi \left(J\right)=0\right\},$$

where ψ(J)=min{ϕ(K):J⊆K⊆W}. However, this closed testing procedure involves invoking the partial true discovery procedures exponentially many times for many different sets and significance levels, resulting in a large computational burden. We can alleviate this burden using shortcuts in several steps, as we will discuss below, finally coming to the procedure described in Sections 2 and 3.

### 4.2 First Shortcut: Using Coherence

According to Equation (6), computation of d(S) involves calculating $d_{i}^{\alpha /m_{S}}\left(F_{i}\cap S\right)$ for exponentially many sets. Even if fast algorithms exist for each partial procedure, the computational cost of so many calls would be prohibitive. In this section, we propose a shortcut that uses only $d_{i}^{\alpha /m_{S}}\left(F_{i}\right)$, drastically reducing the number of calls to $d_{i}^{\alpha /m_{S}}\left(F_{i}\cap S\right)$.

The alternative local test based on $d_{i}^{\alpha /m_{S}}\left(F_{i}\right)$ is defined as follows:

(8) $$\tilde{\phi }\left(S\right)=\left\{\begin{array}{cc} 1, & \text{if}\;d_{i}^{\alpha /m_{S}}\left(F_{i}\right)>\left|F_{i}\setminus S\right|\;\text{for at least one}\;1\leq i\leq m,\\ 0, & \text{otherwise}. \end{array}\right.$$

This local test is a shortcut to ϕ in Equation (6) since a hypothesis can be rejected by $\tilde{\phi }$ only if it is rejected by ϕ, as stated by the following lemma. Type I error control of $\tilde{\phi }$ follows by combining this lemma with Lemma 4.1.

Lemma 4.2 $\tilde{\phi }\left(S\right)\leq \phi \left(S\right)$ for all $S\in 2^{W}$, with equality if $S\cap F_{i}=F_{i}$ or $S\cap F_{i}=\emptyset$ for all 1≤i≤m.

From Lemma 4.2, we see that there is no power loss in replacing ϕ by $\tilde{\phi }$ if S is a union of some focus sets. Power loss can be substantial if S is very unlike such a set. The lemma, therefore, suggests that the resulting shortcut is most useful for sets S that are “nearly” unions of focus sets.

A closed testing procedure $\tilde{d}$ can be defined by $\tilde{\phi }$, analogous to (7). This closed testing procedure needs to call each partial procedure $d_{i}^{\alpha /m_{S}}$ for only focus sets, but still for many values of $\alpha /m_{S}$. We will address this issue in the next section.

### 4.3 Second Shortcut: Using Holm

In this section, we construct a further shortcut for the closed testing procedure based on $\tilde{\phi }$, for which it suffices to calculate $d_{i}^{\alpha /h}\left(F_{i}\right)$, i=1,…,m for only a limited number of values of h. Our reasoning follows the principle of Holm (1979), as outlined in Section 2.

Based on $d_{i}^{\alpha /h}$, we define a local test as follows:

(9) $$\bar{\phi }\left(S\right)=\left\{\begin{array}{cc} 1, & \text{if}\;d_{i}^{\alpha /h}\left(F_{i}\right)>\left|F_{i}\setminus S\right|\;\text{for at least one}\;1\leq i\leq m,\\ 0, & \text{otherwise}, \end{array}\right.$$

where h is understood to be the final value of h upon convergence of Algorithm 1. The following lemma states that $\bar{\phi }$ is a shortcut testing to $\tilde{\phi }$.

Lemma 4.3 $\bar{\phi }\left(S\right)\leq \tilde{\phi }\left(S\right)\;\text{for all}\;S\in 2^{W}$.

We may note that the power loss of $\bar{\phi }\left(S\right)$ is negligible when the number of focus sets that have an empty intersection with S is close to the number of fully rejected focus sets. Otherwise, the power loss will be large. Moreover, Lemma 4.3 is useful because it allows quick determination of the effective local test. Let $\bar{\psi }\left(J\right)=\min\left\{\bar{\phi }\left(K\right):J\subseteq K\subseteq W\right\}$ be the closed testing procedure, and J is any generic subset of W, we then have

Lemma 4.4 $\bar{\psi }\left(J\right)=\bar{\phi }\left(J\right)$.

It follows from Lemma 4.4 that the shortcut calculates whether $H_{S}$ is rejected in O(m) time, after $d_{1}^{\alpha /h}\left(F_{1}\right),\text{…},d_{m}^{\alpha /h}\left(F_{m}\right)$ have been evaluated.

Based on $\bar{\phi }$, the corresponding closed testing procedure can be defined as $\bar{d}$. We show in the following lemma that the proposed procedure $d^{\left(r\right)}\left(S\right)$ in Section 2 is equivalent to $\bar{d}$, which is a shortcut to the closed testing procedure d in Equation (7).

Lemma 4.5 $\bar{d}\left(S\right)=d^{\left(r\right)}\left(S\right)$ for all $S\in 2^{W}$.

From the two‐step shortcut described above, we see that the computational gaps between the procedure proposed in Section 2 and a fully closed testing procedure cause minimal power loss when the set of interest is a union of disjoint focus sets and the number of focus sets that have a nonempty intersection with the set is equal to that of not completely rejected focus sets. Otherwise, the power loss can be substantial after two gaps.

## 5 Global Test Implementation: newFocus

Although the method we have presented is general, we have a special interest in the application of the method based on partial closed testing procedures that use the global test of Goeman et al. (2004) as its local test. For this, we have derived a variant of the shortcut of Xu, Solari, and Goeman (2023). This latter shortcut is only designed for set‐wise family‐wise error rate (FWER) control, that is, for deciding whether $d_{i}^{\alpha }\left(S\right)>0$ for any S of interest; our novel shortcut is specific for finding $d_{i}^{\alpha }\left(F_{i}\right)$ only. The derivation can be found in the Supporting Information. It is implemented in the R package newFocus (Xu, Solari, and Goeman 2021) on CRAN, which also implements the general procedure of Sections 2 and 3.

## 6 Simulations

### 6.1 Focused Power

In this section, we investigate whether combining partial true discovery guarantee procedures has the expected property that it concentrates power on focus sets at the expense of nonfocus sets. We do this using an artificial data example.

We use n=100 samples and w=1000 features, where the response Y is binary, following from Bernoulli distribution with probability 0.5. We vary the number of truly associated features in (200,500,800), that is, the proportion of true nonnull features varies in (0.2,0.5,0.8). The features $X_{1},\text{…},X_{w}$ were drawn independently from a normal distribution with σ=1. The mean of truly associated features is 0.7 and 0 otherwise. The higher the mean, the stronger the association between X and Y. We calculate the p‐value for every feature by testing the association between the feature and the response using an independent sample *t*‐test.

We choose to create a list of 22 sets, 11 of which are focus sets and the others are nonfocus sets, both with TDP in the range of (0,0.1,0.2,…,1). We construct the sets in a way that the focus sets are overlapped with each other and also with the nonfocus sets. More details about the creation of the sets can be found in the Supporting Information.

For the partial true discovery procedures on the 11 focus sets, we use the closed testing procedure based on Fisher's combinations as local tests, for which a fast shortcut is available (Tian et al. 2023), implemented in the R package sumSome (Vesely and Chen 2021). We combine these partially closed testing procedures in the way described in Section 2, using the greedy algorithm in Section 3. As a competitor, we considered the full closed testing procedure with Fisher's combination local tests.

**FIGURE 1:**
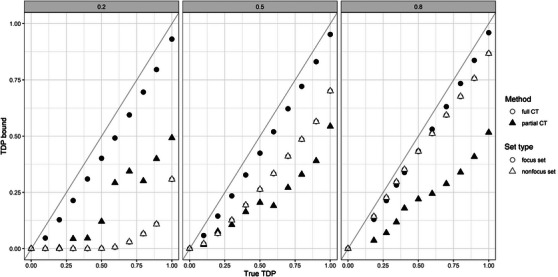
True TDP (*x*‐axis) versus TDP confidence bound (*y*‐axis) by the full closed testing procedure (the empty points) and by partially closed testing (the filled points). The point shape denotes the set type, the circles are focus sets, and the triangles are nonfocus sets. The plots differ by the proportion of true nonnull features.

We calculate TDP on average over 1000 replications for all focus sets and nonfocus sets, summarizing the results in Figure 1. We see in all settings that the proposed procedure gives better TDP bounds for focus sets than the overall Fisher's combinations based closed testing procedure, but not for nonfocus sets, confirming that it concentrates power on focus sets. Moreover, this power is quite stable for different proportions of nonnull features. In contrast, the overall procedure is highly affected by the proportions. Surprisingly, the proposed procedure has also good power on nonfocus sets especially when the true features in the nonfocus sets are very enriched and largely overlapped with the focus sets (see first panel in Figure 1).

### 6.2 DAG‐ and Tree‐Structured Hypotheses

In this work, we do not make any assumptions about the structure of the feature sets. There have been many methods proposed for tree‐structured hypotheses (Blanchard, Neuvial, and Roquain 2020; Bogomolov et al. 2021; Miecznikowski and Wang 2023) or specifically DAG‐structured hypotheses (Guo, Lynch, and Romano 2018; Meijer and Goeman 2015). To better understand the pros and cons of the method, we compare our method with “sanssoucci” (Blanchard, Neuvial, and Roquain 2020) and “DAG” (Meijer and Goeman 2015) for tree‐ and DAG‐structured feature sets. In addition, we compare to Simes‐based closed testing on these feature sets (Goeman et al. 2019).

The artificial data are generated from the R package sanssouci. The complete dyadic tree structure with 100 elements and eight layers is created. The proportion of true nonnulls is set to (0.1,0.2,0.5,0.8), and we consider two situations of nonnulls in the tree structure: (1) they are grouped in one branch of the tree, or (2) they are randomly distributed from all leaves. The strength of the signals is controlled by μ, for which we set μ=1 as weak signals and μ=4 as strong signals. The detailed setting can be found in the Supporting Information.

We calculate the TDP bound of the set of all nonnulls, which is not a node in the tree. The result from R package sanssouci is represented by “tree,” and the result from R package cherry is represented by “dag.” The procedures “ptd@4” and “ptd@all” represent the combined procedures for which the focus sets are nodes at layer 4 of the tree and all nodes of the tree, respectively. The partial true discovery procedure is chosen as a partially closed testing procedure, for which the local test is Fisher's combination test, which is also the test method chosen for the dag method.

**FIGURE 2:**
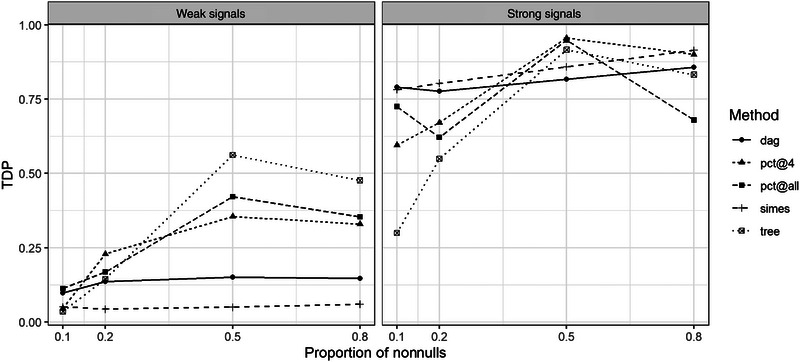
TDP bound for the set of all true nonnulls for grouped true nonnull signals in the tree.

The mean TDP based on 100 replications is summarized in Figure 2 for grouped true nonnulls. The result for ungrouped true nonnulls are presented in the Supporting Information. It is shown in Figure 2 that “ptd@4” and “ptd@all” are powerful for strong signals as well as the dag and Simes method. The tree method is, however, powerful when the signals are weak, whereas the other methods are less powerful.

## 7 Replicability Analysis With Artificial Data

In recent years, there has been a considerable debate about the lack of replicability of results in many scientific fields (Ioannidis 2005; Nuzzo 2014). This debate has brought about the development of formal statistical methods for assessing replicability (Benjamini and Heller 2008; Friston, Penny, and Glaser 2005; Jaljuli et al. 2023; Wang et al. 2022). Replicability analysis aims to identify the findings that are replicated across independent studies that examine the same features.

Consider the problem where m hypotheses are tested in v studies. Define an m×v matrix of hypotheses ${\left(H_{ij}\right)}_{m\times v}$, with one row per feature and one column per study, where $H_{ij}$ specifies no effect for the ith feature in the jth study. Suppose that we have a p‐value matrix ${\left(p_{ij}\right)}_{m\times v}$ for the hypotheses ${\left(H_{ij}\right)}_{m\times v}$ and assume that $p_{i1},\text{…},p_{iv}$ are independent for each 1≤i≤m and that $p_{ij}$ is stochastically larger than uniform when $H_{ij}$ is true. Let $p_{i(1)}\leq \cdots \leq p_{i(v)}$ be the sorted values of $p_{i1},\text{…},p_{iv}$ for each 1≤i≤m.

The partial conjunction (PC) testing framework of Benjamini and Heller (2008) aims at establishing that an effect was discovered in at least r of v studies, where r is a prespecified integer indicating the minimal replicability requirement. Let $H_{i}^{r/v}$ be the PC hypothesis specifying that fewer than r studies have shown a true effect for the ith feature for 1≤i≤m. Rejecting the PC hypothesis, $H_{i}^{r/v}$ guarantees that the signal in the ith feature has been replicated at least r times across v studies.

A recent proposal for simultaneous testing of the PC hypotheses is AdaFilter (Wang et al. 2022), a multiple testing procedure that increases power by filtering out PC hypotheses that are likely true nulls by using the filtering p‐value $R_{i}=\left(v-r+1\right)p_{i(r-1)}$. Specifically, for a prespecified level α, AdaFilter rejects $H_{i}^{r/v}$ if the selection p‐value $S_{i}=\left(v-r+1\right)p_{i(r)}<\hat{\alpha }$, where $\hat{\alpha }=\sup\left\{\gamma \in \left[0,\alpha \right]:\gamma \sum_{i=1}^{m}1_{\left\{R_{i}<\gamma \right\}}\leq \alpha \right\}$. Wang et al. (2022) proved that the AdaFilter procedure controls the family‐wise error at level α under the assumption that all m×vp‐values are independent.

A more flexible approach to replicability analysis advocated by Heller (2011) and Jaljuli et al. (2023) is based on simultaneous testing PC hypotheses $H_{i}^{r/v}$ for all 1≤i≤m and all 1≤r≤v, thereby adding another layer of multiplicity. Importantly, PC testing for all possible values of r provides a lower bound d for the number of studies that replicated, and it allows to make simultaneous statements such as “with 95% confidence, out of v studies, at least $d_{A}=2$ studies shown an effect for feature A, at least $d_{B}=4$ for feature B, no one ($d_{C}=0$) for feature C, etc.” compared to fixed‐r PC testing, which simply identifies the features that shown an effect in at least r studies.

We implemented this alternative approach by combining partially closed testing procedures. Define as focus sets the partition $F=\{F_{1},\text{…},F_{m}\}$ of the w=m×v hypotheses, with $F_{i}$ representing the ith feature by including the indexes corresponding to the hypotheses $H_{i1},\text{…},H_{iv}$. We derive the lower bound $d_{i}=d_{i}^{\alpha /m}\left(F_{i}\right)$ for each of the focus sets by closed testing based on Fisher's combination method as a local test (Goeman and Solari 2011). Computation of the lower bound $d_{i}$ amounts to test in order $H_{i}^{r/v}$ for increasing values of r, which provides $d_{i}=\max\left\{r:p_{i}^{u/v}\leq \alpha /m\;\;\mathrm{for}\;\;u=0,\text{…},r\right\}$, where $p_{i}^{u/v}$ is the p‐value testing $H_{i}^{u/v}$ by Fisher's combination method (Heller 2011). Closed testing offers simultaneous $d_{i}^{\alpha /m}\left(I\right)$ not only for $I=F_{i}$ but also for any subset of studies $I\subseteq F_{i}$. The algorithm in the R package sumSome provides calculation of $d_{i}^{\alpha /m}\left(I\right)$ with log‐linear complexity in the total number of hypotheses.

We performed a simulation study at level α=5% with m=10 features and v=10 studies. Let $v_{1}$ be the true number of studies with signal. For the first half of features, we vary $v_{1}$ from 0 to v, and for the second half of features, we set $v_{1}$ to zero. We generated independent p‐values following Beta (α,1) or Uniform(0,1) distribution according to the presence or absence of an effect, respectively. We compared partially closed testing with the AdaFilter‐r method for different values of r. AdaFilter‐r lower bound for the ith feature is equal to r if $H_{i}^{r/v}$ is rejected by AdaFilter‐r and 0 otherwise. For each value of $v_{1}$, we calculated the average lower bound of true studies over all features by partially closed testing and AdaFilter‐r methods, for 2≤r≤v. For comparison, the true mean of studies with signal is $\bar{r}=v_{1}/2$. Results average over 1000 replications are reported in Table 2. It is shown that the partially closed testing procedure outperforms AdaFilter in general, with the exception that the prespecified r of AdaFilter is equal to $v_{1}$, where AdaFilter concentrates power on detecting if there are at least r studies showing effects for the first half of features.

**TABLE 2:** Mean lower bound obtained by partial closed testing and AdaFilter‐r methods.

	Number of studies with signal $v_{1}$										
	0	1	2	3	4	5	6	7	8	9	10
true mean $\bar{r}$	0.00	0.50	1.00	1.50	2.00	2.50	3.00	3.50	4.00	4.50	5.00
Partial CT	0.00	0.38	0.83	1.29	1.78	2.28	2.79	3.32	3.85	4.40	4.96
AdaFilter‐2	0.00	0.00	0.95	0.99	1.00	1.00	1.00	1.00	1.00	1.00	1.00
AdaFilter‐3	0.00	0.00	0.01	1.38	1.49	1.49	1.50	1.50	1.50	1.50	1.50
AdaFilter‐4	0.00	0.00	0.00	0.02	1.83	1.99	1.99	2.00	2.00	2.00	2.00
AdaFilter‐5	0.00	0.00	0.00	0.00	0.02	2.26	2.48	2.49	2.50	2.50	2.50
AdaFilter‐6	0.00	0.00	0.00	0.00	0.00	0.02	2.70	2.98	2.99	3.00	3.00
AdaFilter‐7	0.00	0.00	0.00	0.00	0.00	0.00	0.03	3.14	3.48	3.50	3.50
AdaFilter‐8	0.00	0.00	0.00	0.00	0.00	0.00	0.00	0.03	3.66	3.98	4.00
AdaFilter‐9	0.00	0.00	0.00	0.00	0.00	0.00	0.00	0.00	0.04	4.16	4.49
AdaFilter‐10	0.00	0.00	0.00	0.00	0.00	0.00	0.00	0.00	0.01	0.05	4.79

## 8 Discussion

We have shed light on a true discovery guarantee procedure for all possible feature sets, which can specifically focus power on some feature sets of interest but may cost power for other feature sets. The procedure is proved to be a shortcut to the closed testing procedure, for which we show where the potential computational gaps are.

Our method is useful to measure the effect size of the feature set by global testing, which is superior to the original focus‐level procedure that only shows the absence or presence of true features. However, limitations of the method exist, that is, we may lose power when inferring the nonfocus sets and applying the shortcuts. We may shed light on how to improve both the power and computational efficiency in the future study.

The procedure is derived from Bonferroni‐ or Holm‐based combinations of partial procedures. We have shown in the Supporting Information that the overlapping focus sets result in a more conservative procedure than the disjoint focus sets. The power loss can be 50% around in our numerical study, which suggests users choose disjoint focus sets in practice as much as possible.

## Conflicts of Interest

The authors declare no conflicts of interest.

## Open Research Badges

This article has earned an Open Data badge for making publicly available the digitally‐shareable data necessary to reproduce the reported results. The data is available in the Supporting Information section.

This article has earned an open data badge “Reproducible Research” for making publicly available the code necessary to reproduce the reported results. The results reported in this article could fully be reproduced.

## Supporting information

Supporting Information

Supporting Information

## Data Availability

The real data examples that support the findings of this study are openly available in GEO at https://www.ncbi.nlm.nih.gov/geo/query/acc.cgi?acc=GSE68086, reference number [GSE68086].
