# Supplementary material for: Combining Partial True Discovery Guarantee Procedures
Source: Biom J. 2024 Jul 2;66(5):e202300075. doi: 10.1002/bimj.202300075 (PMC12859535; doi:10.1002/bimj.202300075)
Supplement: Supplementary file 1 — Supporting Information [file BIMJ-66-e202300075-s002.pdf]

Biometrical Journal **52** (2010) 61, zzz–zzz / DOI: 10.1002/bimj.200100000

Supporting Information for "Combining partial true discovery guarantee procedures"

Ningning Xu[*,1], **Aldo Solari** [2], and **Jelle J. Goeman** [1]

[1] Department of Biomedical Data Sciences, Leiden University Medical Center, Einthovenweg 20, 2333ZC Leiden, The Netherlands

[2] Department of Economics, Management and Statistics, University of Milano-Bicocca, Piazza dell'Ateneo Nuovo, 1, 20126 Milan, Italy
Department of Economics, Ca' Foscari University of Venice, Cannaregio 873, 30121 Venice, Italy

Received zzz, revised zzz, accepted zzz

1 Proof of Lemma 3.1

Lemma 3.1 $\hat{d}^{(r)}(S) \leq d^{(r)}(S)$ for all $S \subseteq W$.

Proof. Without loss of generality, let $F_1, \cdots, F_m$ be the descending order of $d_i^{\alpha/h}(S)$. We then have

$$\begin{aligned}
d^{(r)}(S) &\geq d^{(r-1)}(F_1) - |F_1 \setminus S| + d^{(r-1)}(S \setminus F_1) \\
&\geq d^{(r-1)}(F_1) - |F_1 \setminus S| + d^{(r-2)}(F_2) - |F_2 \setminus (S \setminus F_1)| + d^{(r-2)}(S \setminus F_1 \setminus F_2) \\
&\geq d^{(r-1)}(F_1) - |F_1 \setminus S| + \cdots + d^{(r-m)}(F_m) - |F_m \setminus (S \setminus \bigcup_{i=1}^{m-1} F_i| + d^{(r-m)}(S \setminus \bigcup_{i=1}^{m} F_i) \\
&\geq d_1^{\alpha/h} - |F_1 \setminus S| + \cdots + d_m^{\alpha/h} - |F_m \setminus (S \setminus \bigcup_{i=1}^{m-1} F_i| + 0 \text{ (monotonicity of interpolation)} \\
&= \hat{d}^{(r)}(S)
\end{aligned}$$

□

2 Proof of Lemma 4.1

Lemma 4.1 If $P \in H_S$, then $P(\phi(S) = 1) \leq \alpha$.

Proof. Let $P \in H_S$. Call $M_S = \{i\colon F_i \cap S \neq \emptyset\}$, i.e. the collection of focus sets that have non-empty intersection with S, thereby $|M_S| = m_S$. We remark the equivalence between the two events: (1) there is at least one positive $d_i^{\alpha/m_S}(F_i \cap S)$ for $1 \leq i \leq m$ and (2) $\sum_{i \in M_S} d_i^{\alpha/m_S}(F_i \cap S)$ is positive. We

*Corresponding author: e-mail: n.xu@lumc.nl

© 2010 WILEY-VCH Verlag GmbH & Co. KGaA, Weinheim

therefore have that

$$\begin{aligned}P(\phi(S)=1) &= P(\sum_{i\in M_S} d_i^{\alpha/m_S}(F_i\cap S)>0)\\ &\le \sum_{i\in M_S} P(d_i^{\alpha/m_S}(F_i\cap S)>0)\\ &\le \sum_{i\in M_S}\frac{\alpha}{m_S}\\ &= \alpha,\end{aligned}$$

where the second inequality uses Equation (2) in the main text, and the fact that $|W_1 \cap S| = 0$ when $P \in H_S$. □

3 Proof of Lemma 4.2

Lemma 4.2 $\tilde{\phi}(S) \le \phi(S)$ for all $S \in 2^W$, with equality if $S\cap F_i = F_i$ or $S\cap F_i = \emptyset$ for all $1 \le i \le m$.

P r o o f. The first statement of this Lemma follows immediately from the coherence property of closed testing in Lemma 3 and 4 of Goeman et al. [2021], which says that $d_i^{\alpha/m_S}(F_i\cap S) \ge d_i^{\alpha/m_S}(F_i) - |F_i \setminus S|$, since $F_i\cap S$ and $F_i \setminus S$ are disjoint. Therefore, $d_i^{\alpha/m_S}(F_i) - |F_i\setminus S| > 0$ implies that $d_i^{\alpha/m_S}(F_i\cap S) > 0$.

Suppose that for all $i = 1,\ldots,m$ either $S\cap F_i = F_i$ or $F_i \cap S = \emptyset$ holds and that $\tilde{\phi}(S) = 0$. Choose any $1 \le i \le m$. If $S \cap F_i = F_i$, we have

$$d_i^{\alpha/m_S}(S\cap F_i) = d_i^{\alpha/m_S}(F_i) \le |F_i\setminus S| = 0;$$

if $F_i \cap S = \emptyset$, we have

$$d_i^{\alpha/m_S}(S\cap F_i) = d_i^{\alpha/m_S}(\emptyset) = 0.$$

Since $d_i^{\alpha/m_S}(S \cap F_i) = 0$ for all $1 \le i \le m$, we have $\phi(S) = 0$. This proves the second statement. □

4 Proof of Lemma 4.3

Lemma 4.3 $\bar{\phi}(S) \le \tilde{\phi}(S)$ for all $S \in 2^W$.

P r o o f. Suppose that $\bar{\phi}(S) = 1$, which implies that there exists a focus set F_i for which $d_i^{\alpha/h}(F_i) > |F_i \setminus S|$. We will show that $\tilde{\phi}(S) = 1$. We consider separately the cases that $m_S \le h$ and $m_S > h$.

First, suppose $m_S \le h$, we have $\alpha/m_S \ge \alpha/h$ so as to $d_i^{\alpha/m_S}(F_i) \ge d_i^{\alpha/h}(F_i) > |F_i \setminus S|$, thereby $\tilde{\phi}(S) = 1$ by Equation (7) in the main text.

Next, suppose $m_S > h$. Let $m = h_0 > h_1 > \cdots > h_k = h$ be the steps of the Holm's factor update in Algorithm 1. Since $m \ge m_S > h$, there is a step j such that $h_j \ge m_S > h_{j+1}$. By definition of h_{j+1}, we have $|\{i : d_i^{\alpha/h_j}(F_i) = |F_i|\}| = m - h_{j+1}$. Since $m_S \le h_j$, we have $d_i^{\alpha/m_S}(F_i) \ge d_i^{\alpha/h_j}(F_i)$ so that $|\{i : d_i^{\alpha/m_S}(F_i) = |F_i|\}| \ge m - h_{j+1} > m - m_S$. This means that the number of fully rejected focus sets at level α/m_S is at least as large as the number of focus sets that have non-empty intersection with S. Therefore, there must exist at least one focus set F_j, for which $d_j^{\alpha/m_S}(F_j) = |F_j|$ and $F_j \cap S \neq \emptyset$. Consequently, $d_j^{\alpha/m_S}(F_j) - |F_j \setminus S| = |F_j| - |F_j \setminus S| > 0$ so to $\tilde{\phi}(S) = 1$. □

© 2010 WILEY-VCH Verlag GmbH & Co. KGaA, Weinheim

5 Proof of Lemma 4.4

Lemma 4.4

$$\bar{\psi}(J) = \min\{\bar{\phi}(K) : J \subseteq K \subseteq W\} = \bar{\phi}(J).$$

Proof. Let $\bar{\phi}(J) = 1$ and $K \supseteq J$. Then there exists $1 \leq i \leq m$ such that

$$d_i^{\alpha/h}(F_i) > |F_i \setminus J| > |F_i \setminus K|,$$

so $\bar{\phi}(K) = 1$. Since $K \supseteq J$ was arbitrary, we have $\bar{\psi}(J) = 1$. If $\bar{\phi}(J) = 0$, then $\bar{\psi}(J) = 0$ by definition. □

6 Proof of Lemma 4.5

Lemma 4.5 $\bar{d}(S) = d^{(r)}(S)$ for all $S \in 2^W$.

Proof. we first prove that $\bar{\phi}(S) = 1$ if and only if $d^{(r)}(S) > 0$.

Suppose that $\bar{\phi}(S) = 1$, i.e. there exists an F_i such that $d_i^{\alpha/h} - |F_i \setminus S| > 0$, so that $d^{(r)}(S) \geq d^{(1)}(S) \geq d_i^{\alpha/h}(F_i) - |F_i \setminus S| + d_h^{(0)}(S \setminus F_i) > 0$. Now $d^{(r)}(S) \geq \bar{d}(S)$ follows immediately from Corollary 1 in Goeman et al. [2021].

To show the converse, $d^{(r)}(S) \leq \bar{d}(S)$, we use induction. We have that $d^{(0)}(S) \leq \bar{d}(S)$. Suppose that $d^{(i)}(S) \leq \bar{d}(S)$. Taking the interpolation on both sides preserves the relationship. The interpolation of $d^{(i)}(S)$ is $d^{(i+1)}(S)$; the interpolation of $\bar{d}(S)$ is $\bar{d}(S)$ by [Goeman et al., 2021, Lemma 4]. Therefore, $d^{(i+1)}(S) \leq \bar{d}(S)$, as was to be shown. □

7 Simulations

7.1 Focused power

We associate Y and X in the following way:

$$X[Y == 1, \text{true non-nulls}] = X[Y == 1, \text{true non-nulls}] + 0.7,$$

that is, for the observations where $Y = 1$, the mean of the truly associated features is 0.7 and is 0 otherwise. This value controls the strength of association between Y and true features. The higher the value is, the strong association between Y and X.

We choose to create a list of 22 feature sets, 11 of which are focus sets and the remaining are non-focus sets, both with TDP in the range of (0, 0.1, 0.2, $\cdots$, 1). For better understanding of the creation, we illustrate the process in Table 7.1. The sets with zero TDP are always the set of all null features. For the sets with non-zero TDP, we first split all true features into 10 disjoint groups in such a way that the groups are increasing in size so that sets with high TDP have high number of true features, that is, $g_i \cap g_j = \emptyset$ for $i \neq j$ and $|g_1| \leq \cdots \leq |g_{10}|$. To obtain the overlapped sets, we merge the neighboring two groups into one group, $t_1, \cdots, t_{10}$, which will be used as true features in the set. To achieve a desired TDP, we then randomly choose a number of null features, n_i, which can be calculated from $TDP = \frac{|t_i|}{|t_i|+|n_i|}$. We randomly repeat the above steps twice to get the focus and non-focus sets with non-zero TDP. The detailed R code can be found in the data and coding supplement.

© 2010 WILEY-VCH Verlag GmbH & Co. KGaA, Weinheim

Table 1 Steps to generate focus and non-focus sets.

	TDP					
steps	0	0.1	0.2	$\cdots$	0.9	1
1-disjoint groups		g_1	g_2	$\cdots$	g_9	g_{10}
2-true feature sets		$t_1 = g_1 \cup g_2$	$t_2 = g_2 \cup g_3$	$\cdots$	$t_9 = g_9 \cup g_{10}$	$t_{10} = g_{10}$
3-null feature sets		n_1	n_2	$\cdots$	n_9	n_{10}
4-final sets		$t_1 \cup n_1$	$t_2 \cup n_2$	$\cdots$	$t_9 \cup n_9$	$t_{10} \cup n_{10}$

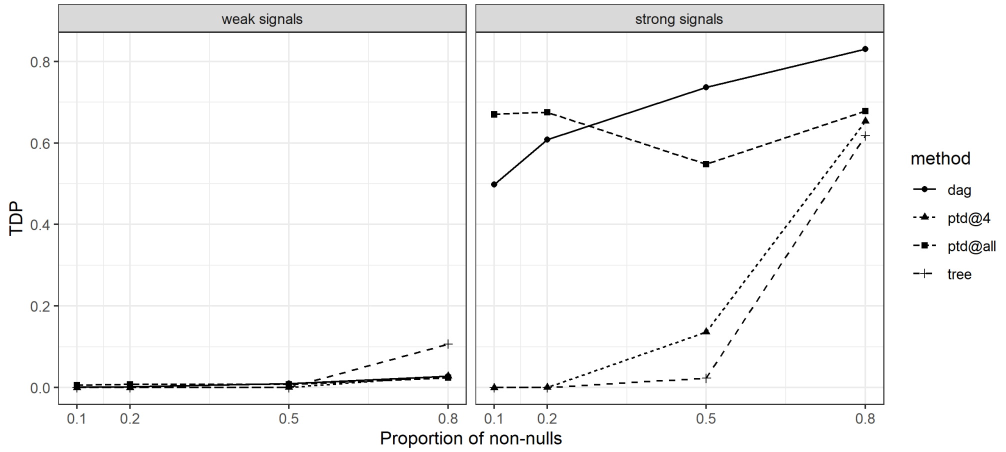

Figure 1 TDP bound for the set of all true non-nulls, for ungrouped true non-null signals in the tree.

7.2 DAG and tree structured hypotheses

The artificial data is generated from R package `sanssouci`. We use *dyadic.from.height* function to create a complete dyadic a complete dyadic tree structure with 100 elements and 8 layers. The *gen.mu.leaves* is used to get the strong signals for $\mu = 4$ and the weak signals for $\mu = 1.5$. The p-values are then obtained by *gen.p.values* based on the μ values from *gen.mu.leaves*.

We present the result for ungrouped true non-nulls in Figure 1, where we see the consistent result with Figure 2 in the main text, that is, the tree method is more powerful than others when the signals are weak and it is less powerful than others when the signals are strong. We also note that the size, number of focus sets influence more for the ungrouped true non-nulls than for the grouped true non-nulls.

7.3 Bonferroni-based local test

We use numerical experiment to show the power loss of Bonferroni-based local test when the focus sets are overlapped with each other.

We use $n = 100$ samples and $w = 1000$ features, where the response Y is binary, following from bernoulli distribution with probability 0.5. We vary the number of truly associated features in $(200, 500, 800)$, i.e. the proportion of true non-null features varies in $(0.2, 0.5, 0.8)$. We then create 20 disjoint focus sets and 20 overlapped focus sets, repsectively, and parial closed testing procedures with Fisher's combination test are applied to get the lower true discovery bounds of the focus set. The TDP of the full feature set is then calculated based on the partial procedures.

We present the result in Figure 7.3, where we see the power loss of overlapped focus sets in contrast with disjoint focus sets. In this example, the loss can be 50% around, we thus suggest to choose disjoint focus sets if possible.

© 2010 WILEY-VCH Verlag GmbH & Co. KGaA, Weinheim

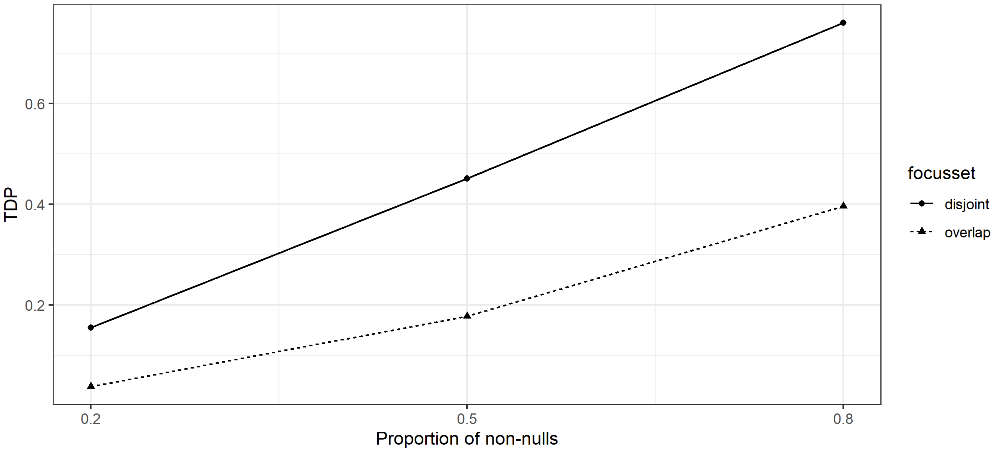

Figure 2 TDP of the full feature set when focus sets are disjoint in contrast with overlapped focus sets.

8 Gene Ontology analysis with real data set

Next, we illustrate the application of our method on gene expression data. When analyzing such data, it is common to focus on gene sets, e.g. from the Gene Ontology (GO) database.

To test the group association between the expression of genes in a gene set and the outcome of interest, such as disease or treatment, globaltest [Goeman et al., 2004] is a popular method, with good power especially when individual gene is associated with the outcome in a weak way. To control the family-wise error rate when testing for all GO terms, Goeman and Mansmann [2008] proposed the "focus level" procedure. This procedure finds an adjusted p-value for each GO term, corresponding to the null hypothesis that the GO term contains no truly associated genes. Instead, we use the combined partial procedures to derive a simultaneous lower confidence bound for the number of truly associated genes in each GO term. Our procedure can be seen as a qualitative improvement to the focus level procedure: instead of just p-values for the presence of any signal, it gives lower confidence bounds for the abundance of the signal.

To use the globaltest in a computationally efficient way, we adapted the shortcut of closed testing with globaltest in Xu et al. [2023] to calculate $d_i^{\alpha/h}$. More detail about this shortcut is given in the Supporting Information. We used this shortcut to calculate the true discoveries in each focus set.

We demonstrate the application of the proposed method with an RNA-seq gene expression data, available in GEO with ID "GSE68086" Best et al. [2015]. This study investigates differential gene expression between 55 healthy individuals and 230 patients with six different malignant tumors. After filtering out genes that are expressed in less than 50% of the samples, we obtained 8138 genes. These genes were mapped to the GO database via the `gtGO` function of `globaltest` package, resulting in 9596 GO terms in the Biological Process sub-ontology. These Go terms were highly overlapping and their sizes ranged from 1 to 6876. The filtered count data was normalized using `voom` function of `limma` package.

As focus sets we chose all 130 GO terms with size between 500 and 1000, a collection of sets with high overlap. The union of these sets covered 94% of the genes present in at least one GO term. The remaining 6% of the genes that are not annotated to any focus sets terms are grouped together as a separate focus set, so that we had in 131 focus sets in total.

We show in Figure 8 the lower confidence bounds for the TDP of all gene sets. Figure 8 confirms that the power of our method concentrates on focus sets rather than non-focus sets. In particular, we see that the TDP of extremely small non-focus sets is often zero, due to the small overlap between these non-focus sets with focus sets.

© 2010 WILEY-VCH Verlag GmbH & Co. KGaA, Weinheim

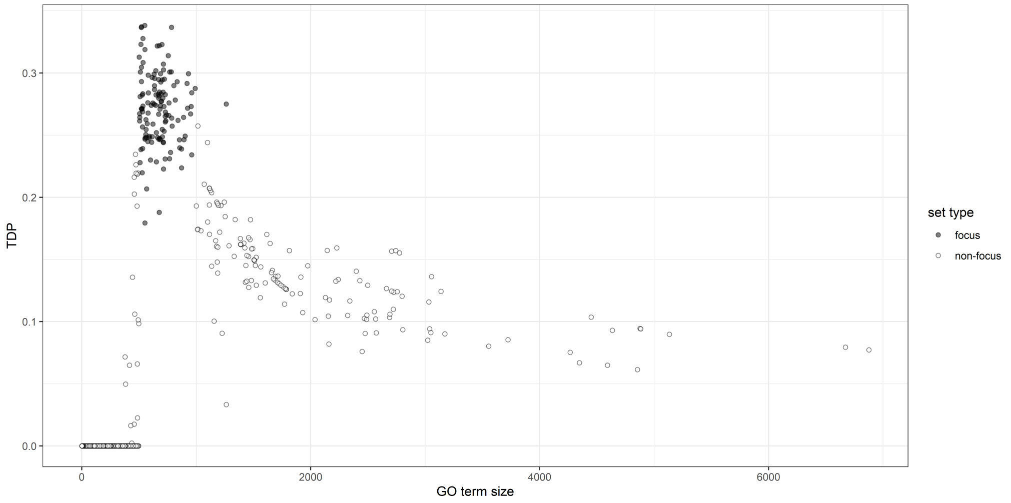

Figure 3 TDP lower confidence bound in GO terms.

9 Shortcut of closed testing with globaltest for true discovery guarantee

We use the same the setup of globaltest as in Xu et al. [2023], where g_R is the observed test statistic and c_R^α is the theoretical $1-\alpha$ critical value for the hypothesis H_R. Under the null hypothesis, the distribution of g_R is asymptotically equivalent to that of a weighted sum of independent χ_1^2 variables, thereby c_R^α is a function of the weights $\boldsymbol{\lambda}_R$. More detail about the calculations of g_R, c_R^α and $\boldsymbol{\lambda}_R$ can be found in Xu et al. [2023].

It is known that closed testing rejects H_R if and only if $g_S \geq c_S$ with $S \supseteq R$, i.e. requiring an exponential number of tests, which is not feasible for large-scale study. Xu et al. [2023] presented an efficient shortcut for testing H_R by the closed testing with globaltest. To calculate $d_i^{\alpha/h}(F_i)$ by the partial closed testing procedure, we have to find out the largest subset of F_i that is not rejected by the globaltest, in terms of Equation (1).

More specifically, we first levelize all subsets of F_i in term of their size. The subsets with same size are at the same level. Then for each level ℓ, we define $g_{min}(\ell)$ and $c_{max}(\ell)$ in the same way as in Xu et al. [2023].If $g_{min}(\ell) > c_{max}(\ell)$, we conclude that all subsets of F_i at level ℓ are rejected. We start from the highest level $|F_i|$ and repeat the comparison between $g_{min}(\ell)$ and $c_{max}(\ell)$ for each level. When $g_{min}(\ell) < c_{max}(\ell)$ occurs, it leads to a “single-step” shortcut in Xu et al. [2023], where we obtain

$$d_i^{\alpha/H}(F_i) \geq |F_i| - \ell.$$

From Xu et al. [2023], we know that there might be an “unsure” situation when $g_{min}(\ell) < c_{max}(\ell)$ and there is a heuristic example, say set V, for which $g_V \geq c_V$ with $|V| = \ell$. In this case, we adopt the branch and bound algorithm to split all subsets into two disjoint spaces, within each $gmin$ and c_{max} are recalculated and compared. For those parts with unsure outcomes, we are allowed to continue branching until a certain outcome obtained or the computation burden exceeds our specified limits. The detailed information of the adapted shortcut is outlined in Algorithm 1.

© 2010 WILEY-VCH Verlag GmbH & Co. KGaA, Weinheim

input : Searching space that includes all subset of F_i: $\mathcal{S} = 2^{F_i}$
output: lower bound of true discoveries in F_i: $d_i^{\alpha/h}(F_i)$
Initialization:
queue $\leftarrow$ ($\mathcal{S}$)
max-not-rejected-example = `Heuristic(`$\mathcal{S}$`)`
iteration = 0
while *queue is empty or iteration exceeds the prespecified size* **do**
  iteration++
  $C \leftarrow$ the first element in queue; remove C from the queue
  if `Bound(`C`)` $\leq$ *max-not-rejected-example* **then**
    Do nothing
  else
    max-not-rejected-example = $\max\{$max-not-rejected-example, `Heuristic(`C`)`$\}$
    Split C into two disjoint subspaces C' and C'' (branching rule)
    Add C' and C'' to queue
  end
end max-not-rejected-level = $\max\{$max-not-rejected-example, $\max_{C \in \text{queue}}\{$`Bound(`$C$`)`$\}\}$
return $|F_i|$ - max-not-rejected-level

Algorithm 1: Calculation of $d_i^{\alpha/h}(F_i)$ by closed testing with globaltest (CTGT)

References

Myron G Best, Nik Sol, Irsan Kooi, Jihane Tannous, Bart A Westerman, François Rustenburg, Pepijn Schellen, Heleen Verschueren, Edward Post, Jan Koster, et al. Rna-seq of tumor-educated platelets enables blood-based pan-cancer, multiclass, and molecular pathway cancer diagnostics. *Cancer cell*, 28(5):666–676, 2015.

Jelle J Goeman and Ulrich Mansmann. Multiple testing on the directed acyclic graph of gene ontology. *Bioinformatics*, 24(4):537–544, 2008.

Jelle J. Goeman, Sara A. van de Geer, Floor de Kort, and Hans C. van Houwelingen. A global test for groups of genes: testing association with a clinical outcome. *Bioinformatics*, 20(1):93–99, 2004.

Jelle J. Goeman, Jesse Hemerik, and Aldo Solari. Only closed testing procedures are admissible for controlling false discovery proportions. *The Annals of Statistics*, 49(2):1218 – 1238, 2021. doi: 10.1214/20-AOS1999. URL `https://doi.org/10.1214/20-AOS1999`.

Ningning Xu, Aldo Solari, and Jelle J Goeman. Closed testing with globaltest, with application in metabolomics. *Biometrics*, 79(2):1103–1113, 2023.

© 2010 WILEY-VCH Verlag GmbH & Co. KGaA, Weinheim

Function `Bound(`$\mathcal{S}$`)`**:**
    $\ell_{top} \leftarrow \max\{|I| : I \in \mathcal{S}\}$
    $\ell_{bottom} \leftarrow \min\{|I| : I \in \mathcal{S}\}$
    for ℓ *in* $\ell_{top} : \ell_{bottom}$ **do**
        The minimum test statistic at level ℓ: $g_{min}(\mathcal{S}, \ell)$
        The maximal critical value at level ℓ: $c_{max}(\mathcal{S}, \ell)$
        if $g_{min}(\ell) < c_{max}(\ell)$ **then**
            return ℓ
            break
        end
    end
end
Function `Heuristic(`$\mathcal{S}$`)`**:**
    $\ell_{top} \leftarrow \max\{|I| : I \in \mathcal{S}\}$
    $\ell_{bottom} \leftarrow \min\{|I| : I \in \mathcal{S}\}$
    Sort $g_i, i \in I \in \mathcal{S}$ in ascending order such that $g_{(1)} \leq \cdots, \leq g_{(\ell_{top})}$
    for ℓ *in* $\ell_{top} : \ell_{bottom}$ **do**
        Choose example subset as $B = \{(1), \cdots, (\ell)\}$
        Calculate g_B and c_B
        if $g_B < c_B$ **then**
            return ℓ
            break
        end
    end
end

Algorithm 2: Functions needed in Algorithm 1

© 2010 WILEY-VCH Verlag GmbH & Co. KGaA, Weinheim
