# Supplementary figures and images for: Combining Partial True Discovery Guarantee Procedures
Source: Biom J. 2024 Jul 2;66(5):e202300075. doi: 10.1002/bimj.202300075 (PMC12859535; doi:10.1002/bimj.202300075)

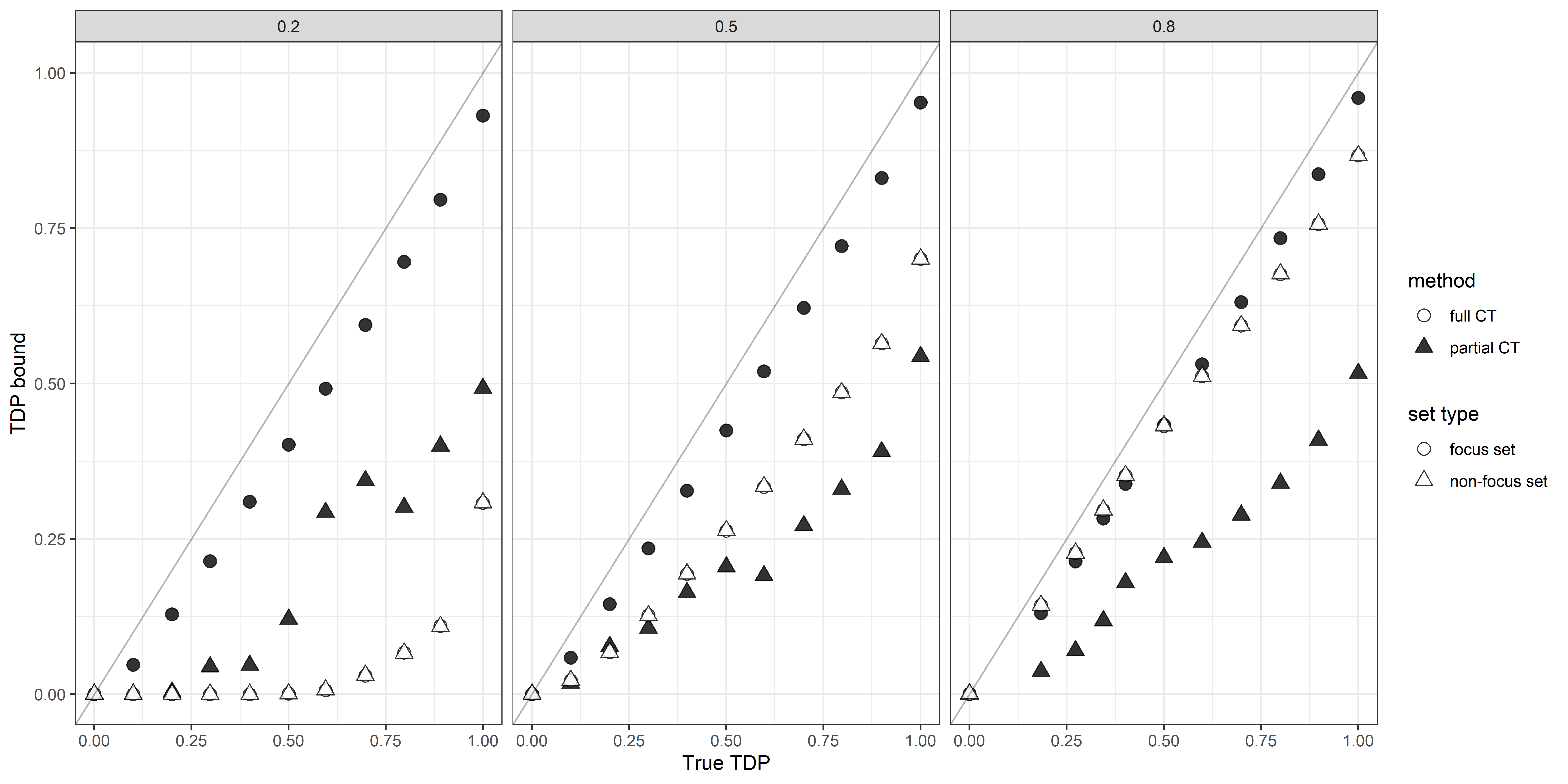

Supplement: Supplementary file 2 — Supporting Information [file BIMJ-66-e202300075-s001.zip › combining_reproduced/ALL_final_results/fig1.png]

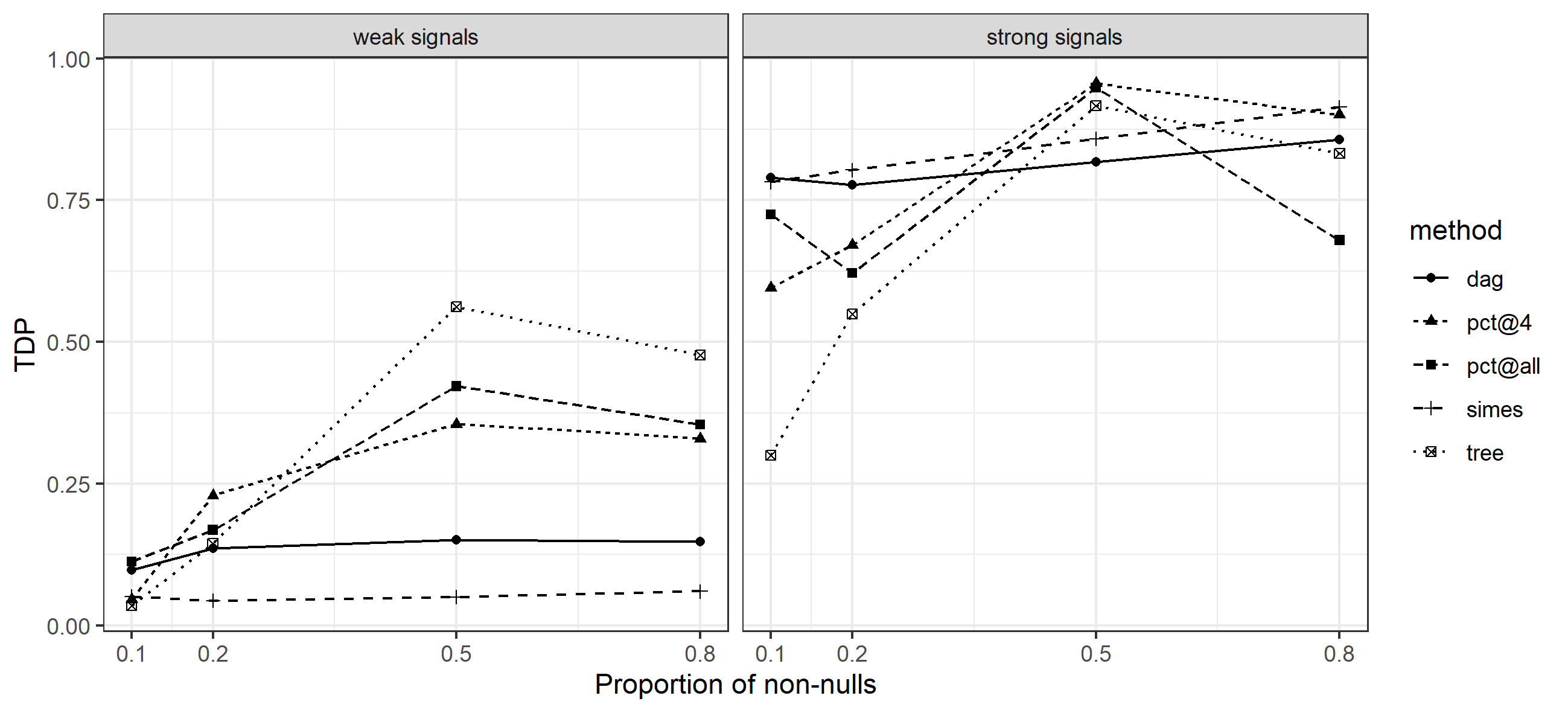

Supplement: Supplementary file 2 — Supporting Information [file BIMJ-66-e202300075-s001.zip › combining_reproduced/ALL_final_results/fig2.png]

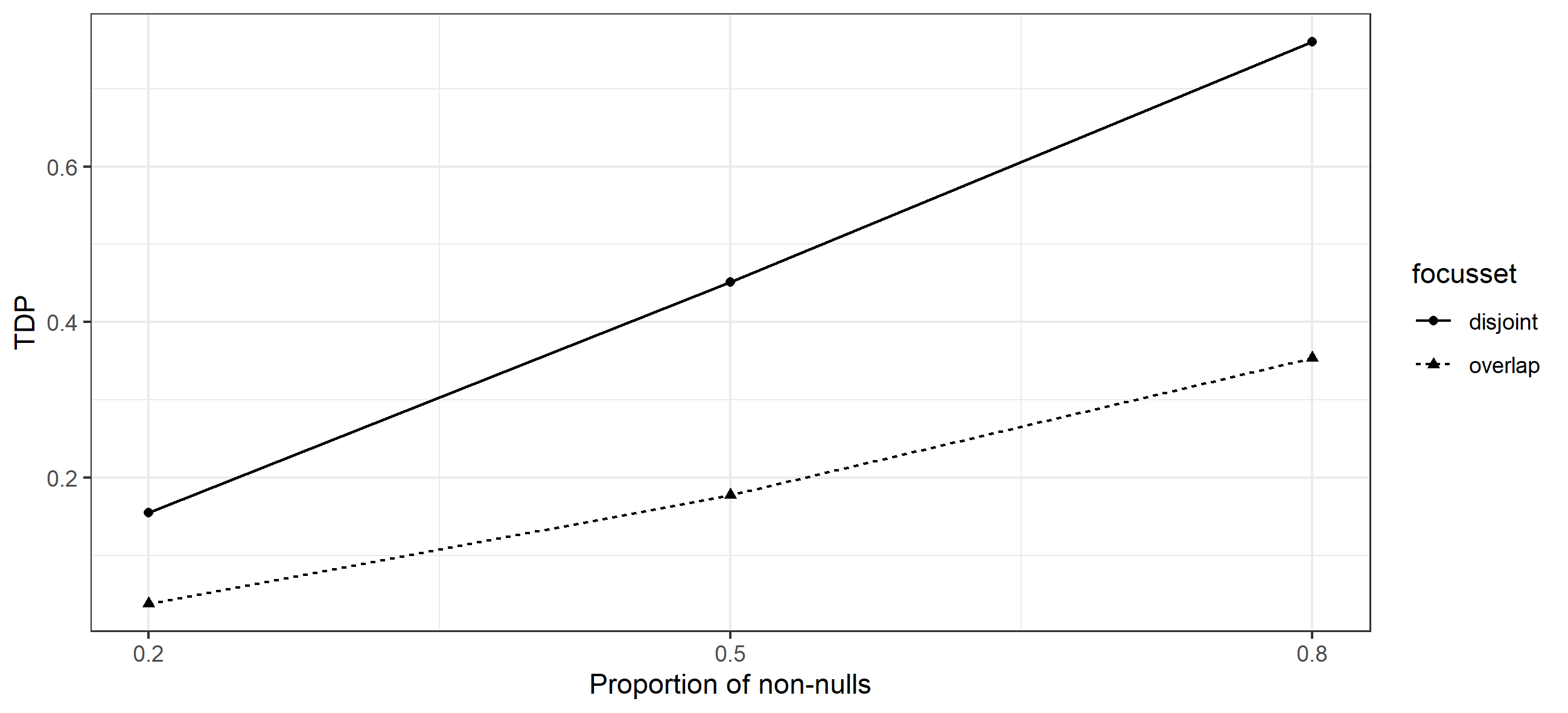

Supplement: Supplementary file 2 — Supporting Information [file BIMJ-66-e202300075-s001.zip › combining_reproduced/ALL_final_results/sfig2.png]

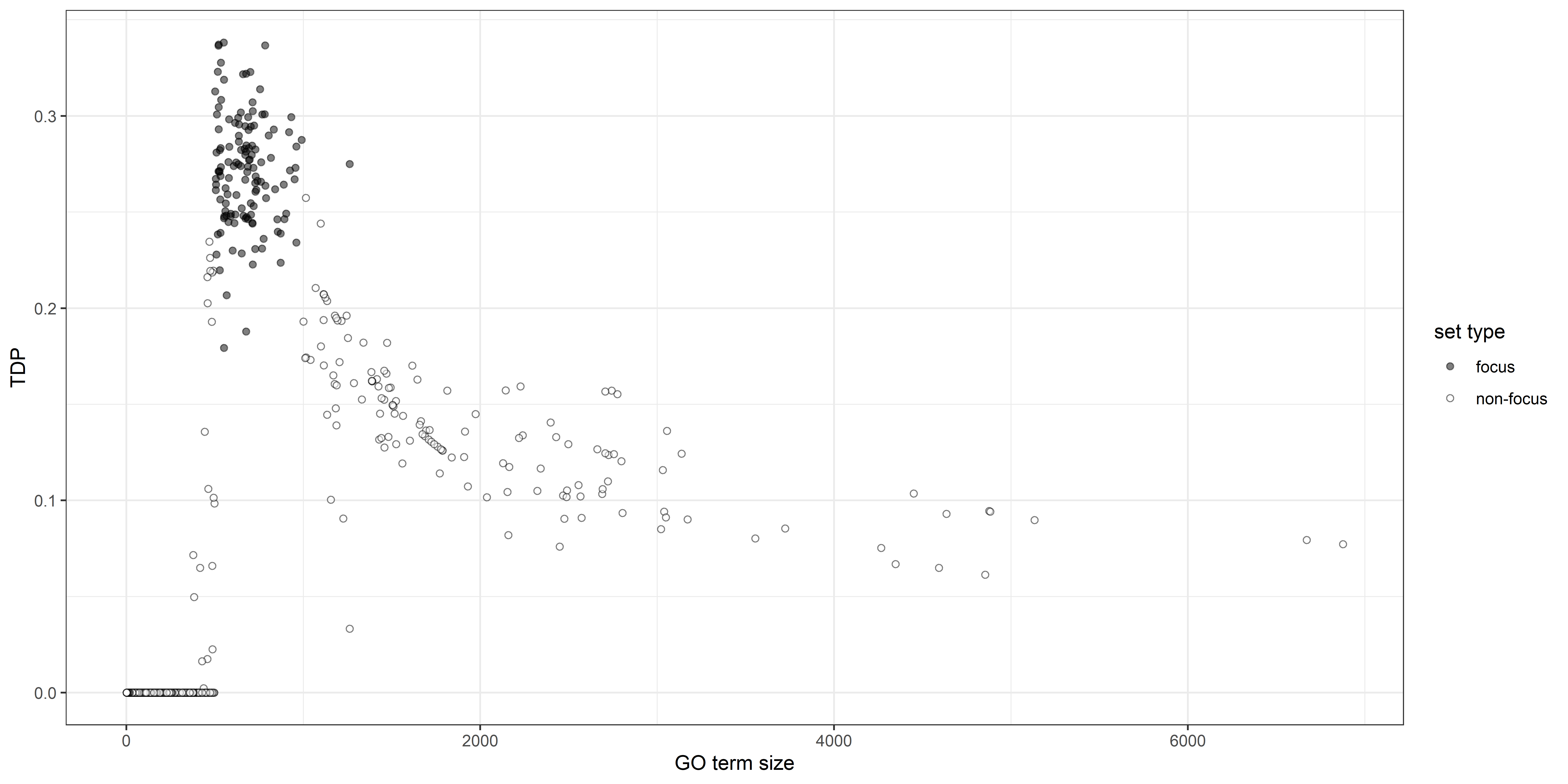

Supplement: Supplementary file 2 — Supporting Information [file BIMJ-66-e202300075-s001.zip › combining_reproduced/ALL_final_results/sfig3.png]

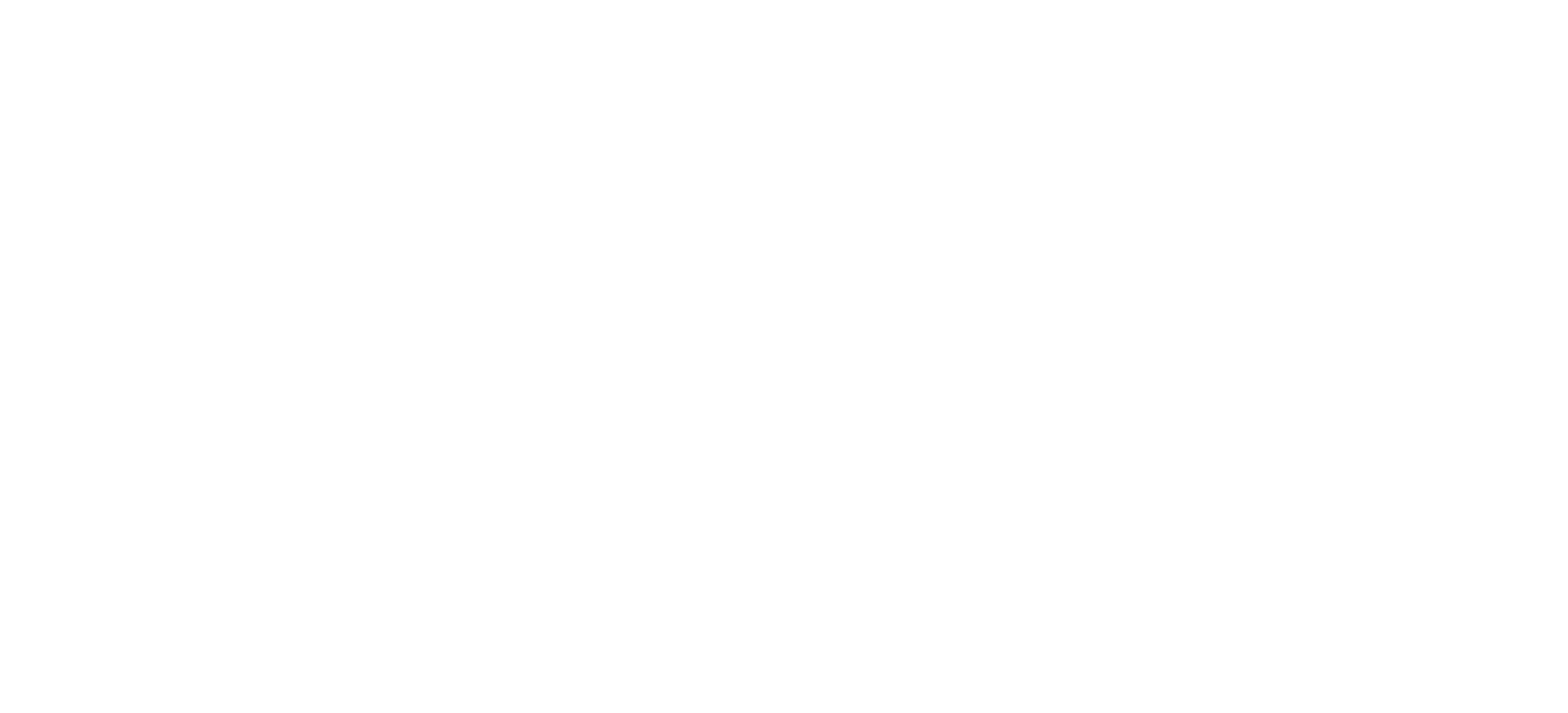

Supplement: Supplementary file 2 — Supporting Information [file BIMJ-66-e202300075-s001.zip › combining_reproduced/ALL_inter_results/grouped/final/grouped_post.png]

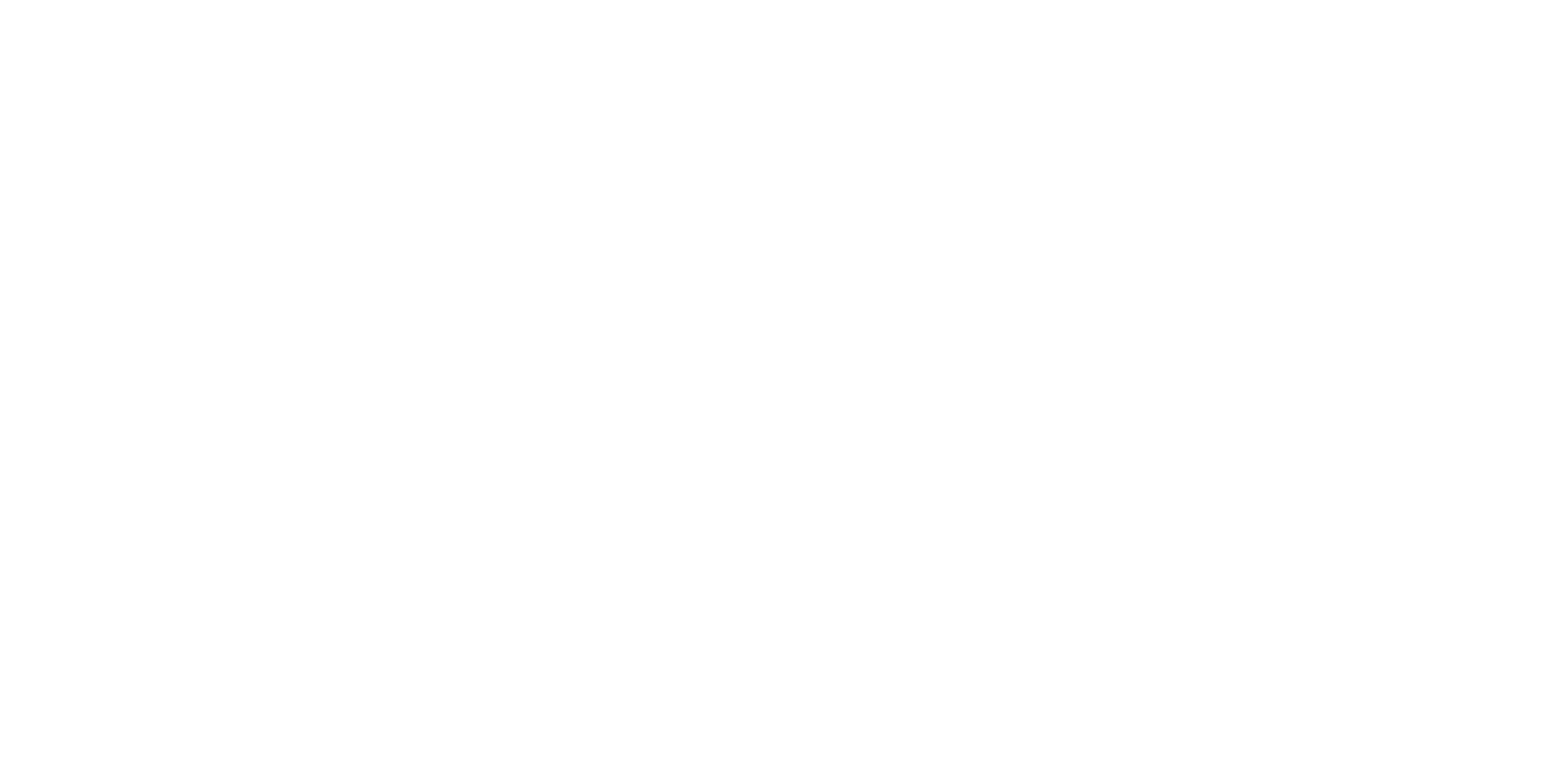

Supplement: Supplementary file 2 — Supporting Information [file BIMJ-66-e202300075-s001.zip › combining_reproduced/real_data/tdp_go.png]

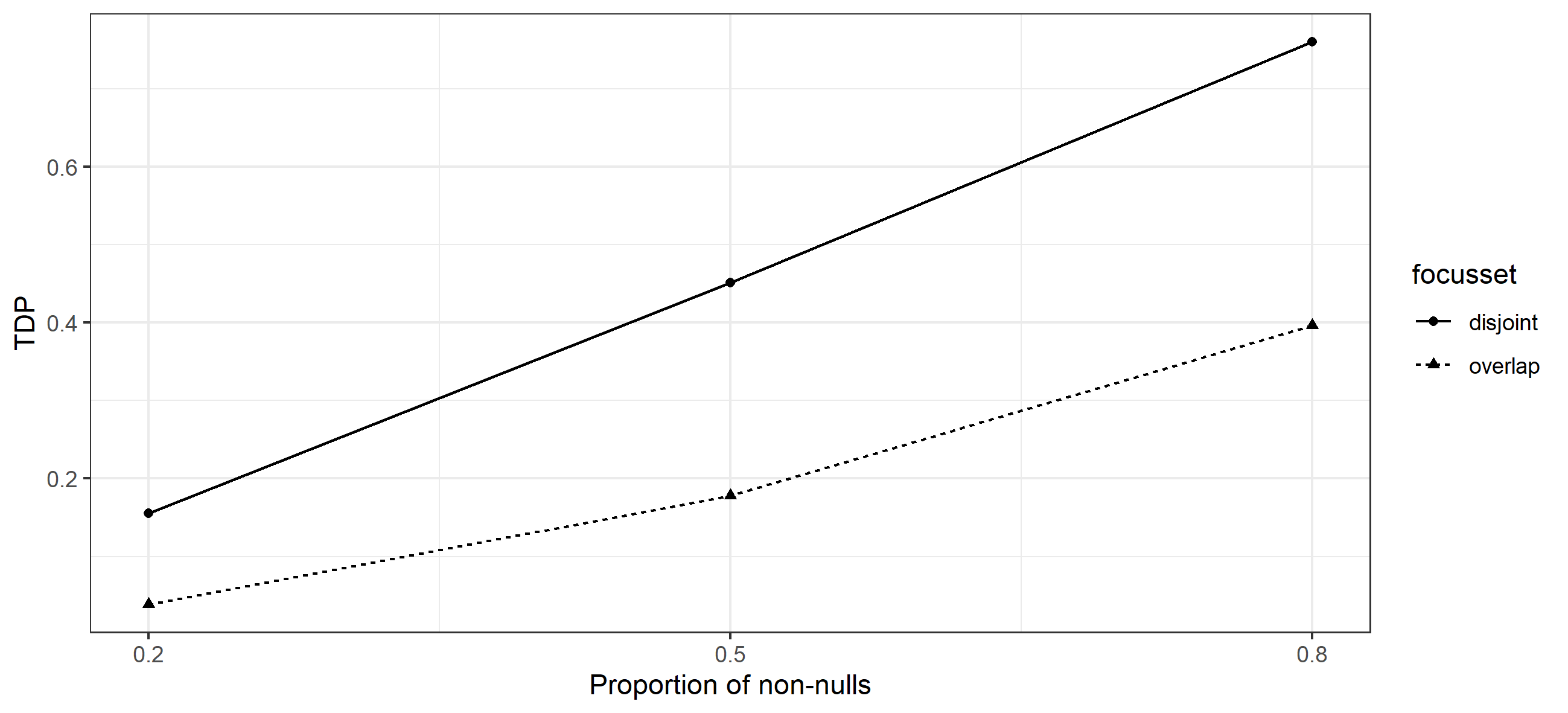

Supplement: Supplementary file 2 — Supporting Information [file BIMJ-66-e202300075-s001.zip › combining_reproduced/simulations/bonferroni_loss-SFig2/boferroni_loss.png]

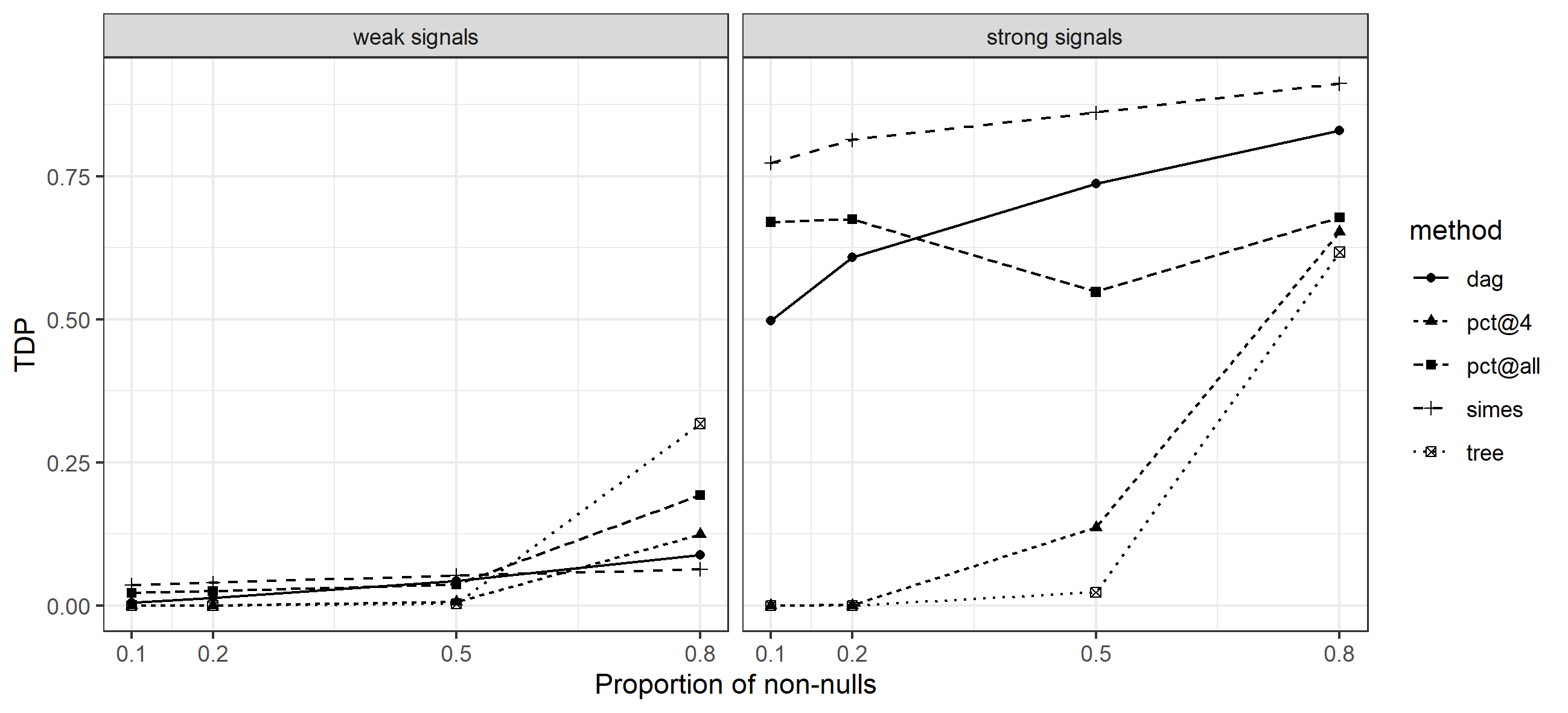

Supplement: Supplementary file 2 — Supporting Information [file BIMJ-66-e202300075-s001.zip › combining_reproduced/simulations/dag_tree_hyps-Fig2/ungrouped/final/ungrouped_post_BW.png]
